# Systematic Review of Artificial Intelligence and Electrocardiography for Cardiovascular Disease Diagnosis

**DOI:** 10.3390/bioengineering12111248

**Published:** 2025-11-14

**Authors:** Hernando Velandia, Aldo Pardo, María Isabel Vera, Miguel Vera

**Affiliations:** 1 Universidad de Pamplona, Pamplona 543050, Colombia; hernando.velandia@unipamplona.edu.co (H.V.); apardo13@unipamplona.edu.co (A.P.); 2 Hospital Universitario Erasmo Meoz, Cúcuta 540004, Colombia; maria.verac@unisimon.edu.co; 3 Facultad de Ciencias Básicas y Biomédicas, Universidad Simón Bolívar, Cúcuta 540004, Colombia; 4 Facultad de Ciencias, Universidad de Los Andes, San Cristóbal 5017, Venezuela

**Keywords:** electrocardiography, artificial intelligence, cardiovascular disease, machine learning, wearable devices, explainable AI, synthetic ECG

## Abstract

Cardiovascular diseases (CVDs) are the leading cause of death globally. Electrocardiograms (ECGs) are crucial diagnostic tools; however, their traditional interpretations exhibit limited sensitivity and reproducibility. This systematic review discusses the recent advances in artificial intelligence (AI), including deep learning and machine learning, applied to ECG analysis for CVD detection. It examines over 100 studies from 2019 to 2025, classifying AI applications by disease type (heart failure, myocardial infarction, and atrial fibrillation), model architecture (convolutional neural networks, long short-term memory, and hybrid models), and methodological innovation (signal denoising, synthetic data generation, and explainable AI). Comparative tables and conceptual figures highlight performance metrics, dataset characteristics, and implementation challenges. Our findings indicated that AI models outperform traditional methods, especially in terms of detecting subclinical conditions and enabling real-time monitoring via wearable technologies. Nonetheless, issues such as demographic bias, lack of dataset diversity, and regulatory hurdles persist. The review concludes by offering actionable recommendations to enhance clinical translation, equity, and transparency in AI-ECG applications. These insights aim to guide interdisciplinary efforts toward the safe and effective adoption of AI in cardiovascular diagnostics.

## 1. Introduction

Cardiovascular diseases (CVDs) continue to be the leading cause of death globally, accounting for over 17.9 million deaths annually, and represent a persistent challenge for public health systems [1,2,3]. Their early and accurate diagnosis is essential for improving patient outcomes and alleviating the healthcare burden imposed by CVDs [4,5]. Electrocardiography remains the most widely used, non-invasive diagnostic tool for detecting a broad range of cardiac abnormalities. However, traditional electrocardiogram (ECG) interpretation is often hampered by inter-observer variability, subjectivity, and limited sensitivity for subtle or early detection of clinical deterioration and disease manifestations [6,7].

Recent advances in artificial intelligence (AI), particularly deep learning (DL) and machine learning (ML), have revolutionized ECG analysis. These technologies enable automated, high-throughput, and highly accurate detection of several cardiac conditions, often outperforming traditional methods in terms of signal-to-noise ratio (SNR), sensitivity, and specificity [8,9,10]. ML approaches for stress detection systems have shown great potential in preventing chronic health conditions by analyzing ECG data collected in free-living environments [11].

Despite these advances, several challenges persist. The generalizability of AI models is often constrained by dataset bias, limited demographic representation, and insufficient clinical validation or real-world testing [7,12]. Furthermore, the “black-box” nature of many DL models raises concerns about transparency, explainability, and clinical trust, which are essential for their safe deployment in healthcare settings [13,14].

Previous reviews have addressed specific aspects of the use of AI in ECG applications, such as arrhythmia detection [15,16], extreme learning machines (ELMs) [17,18], and synthetic data generation [12]. However, there is a lack of studies that provide a comprehensive and integrative synthesis encompassing the full spectrum of clinical applications, signal processing techniques, explainability frameworks, and implementation barriers.

The present review addresses these gaps by:

- Conducting a systematic review based on the Preferred Reporting Items for Systematic Reviews and Meta-Analyses (PRISMA) guidelines [19], synthesizing over 100 studies published between 2019 and 2025 on the applications of AI in ECG analysis, covering a spectrum of cardiovascular conditions, including heart failure (HF), myocardial infarction (MI), atrial fibrillation (AF), and stress or anomaly detection;
- Classifying AI-ECG applications in detail by disease type, model architecture, and methodological approach, highlighting convergences and divergences across the literature;
- Comparing the performances of different AI-based methods through comprehensive tables that identify methodological regularities and shared diagnostic outcomes across studies;
- Critically evaluating persistent challenges such as data bias, signal noise, lack of model explainability, and demographic inequities, while offering actionable recommendations for future research and clinical translation; and
- Incorporating visual and tabular formats of data presentation to facilitate interpretation by clinical and technical audiences.

Through this integrative approach, this review summarizes the current state of the art and identifies unresolved challenges, emerging trends, and practical recommendations for the safe, effective, and equitable implementation of AI-ECG technologies in clinical practice. Prior surveys and perspectives have offered a wealth of insights into the diagnostic potential of AI-based methodologies. For instance, Zhou et al. [20] highlighted recent advances in DL for ECG interpretation, whereas others have evaluated the utility of ML-based diagnostics and the associated challenges [21,22,23]. This systematic review provides a much-needed comprehensive and unified synthesis of all such insights.

## 2. Methods

To guide this review, we formulated the following research question:

Which artificial intelligence algorithms have been applied to electrocardiography for cardiovascular detection, diagnosis, prognosis, or monitoring; what clinical performance do they achieve; what datasets support their development; and what challenges, limitations, ethical considerations, and future directions have been reported?

### 2.1. Selection of Resources

A comprehensive literature search was conducted using electronic databases and manual reference screening. The selection process comprised two stages:

Stage 1—Title/abstract screening: A total of 2042 records were retrieved between 1 January 2019, and 15 March 2025, from four databases: PubMed (n = 1102), Scopus (n = 520), Web of Science (n = 250), and arXiv (n = 170). After the removal of 738 duplicates, 1304 unique records were screened for relevance to AI-ECG applications.

Stage 2—Full-text assessment: After the initial screening, 987 records were excluded. The remaining 317 articles were subjected to full-text review by two independent reviewers, based on predefined inclusion and exclusion criteria. All discrepancies were resolved through discussion or consultation with a third reviewer.

Reasons for exclusion included:

- Absence of ECG data
- Lack of AI or ML methods
- Non-original research (reviews, editorials, and commentaries)
- Animal/in vitro studies
- Insufficient performance or outcome data
- Non-English articles

Following the application of eligibility criteria (Section 2.2), 102 studies were included in the final qualitative synthesis.

### 2.2. Eligibility Criteria

Inclusion criteria:

- Original studies applying any AI, ML, DL, transformer-based, hybrid, or explainable AI (XAI) technique to 12-lead, single-lead, or wearable ECG data in human adults (≥18 years).
- Studies reporting diagnostic or prognostic performance (e.g., area under the curve [AUC], sensitivity, specificity, F-score, C-index, etc.).
- Articles that explicitly describe the datasets used for model training and/or testing.
- Publications available in English or Spanish with full text accessible.

Exclusion criteria:

- Conference abstracts, letters, editorials, reviews, or protocols without full primary data.
- In vivo, in silico, or phantom studies.
- Studies lacking clinical reference standards (including cardiologist adjudication, imaging, catheterization, or follow-up).
- Duplicate publications reporting the same cohort without new methodological or outcome data.

### 2.3. Sources of Data

Literature search was performed in the PubMed/MEDLINE, Scopus, Web of Science, and arXiv databases. The search period spanned from January 2019 to March 2025. The strategy was constructed around Population, Intervention, Comparison, and Outcome elements and tailored to each database. The core syntax was (electrocardiogram OR ECG OR EKG) AND (artificial intelligence OR machine learning OR deep learning OR transformer* OR explainable AI) AND (diagnosis* OR detect* OR predict* OR classifier).

**Figure 1:**
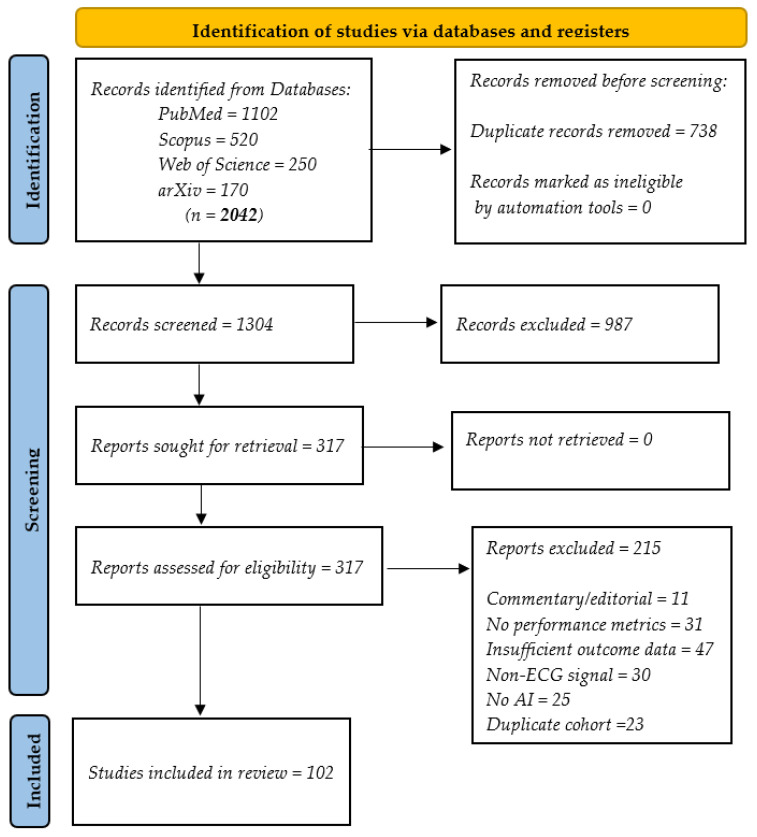
PRISMA 2020 flow diagram. ECG, electrocardiogram; AI, artificial intelligence; PRISMA, Preferred Reporting Items for Systematic Reviews and Meta-Analyses.

Figure 1 summarizes the identification, screening, eligibility, and inclusion phases in accordance with PRISMA guidelines.

## 3. Results

Following the comprehensive search and two-stage screening process outlined in Section 2, a total of 102 primary studies were included in this review. These studies, published between January 2019 and March 2025, encompassed a broad spectrum of AI approaches, ranging from traditional ML classifiers to state-of-the-art DL and XAI models applied to 12-lead, single-lead, and wearable ECG data.

Collectively, these studies incorporated a large volume of ECG tracings obtained from hospital archives, multicenter registries, and publicly available benchmark datasets. The selected studies explored diverse clinical objectives, including:

- Detection of occult structural heart disease,
- Diagnosis of arrhythmias,
- Risk stratification for HF and acute coronary syndromes, and
- Synthetic ECG generation for data augmentation and model training.

To present the findings coherently, the results are organized into four thematic domains:

1. AI-ECG applications across use cases
2. Emerging applications: stress detection, real-life monitoring, and anomaly recognition
3. Synthetic ECG generation using deep generative models (DGMs)
4. Clinical validation studies and implementation challenges

### 3.1. AI-ECG Applications Across Use Cases

The application of AI-based methods to electrocardiographic data has enabled a wide array of use cases across the cardiovascular care continuum. These use cases include disease-specific clinical diagnostics, physiological monitoring, early risk detection in asymptomatic individuals, and real-time decision support through portable and wearable technologies.

**Figure 2:**
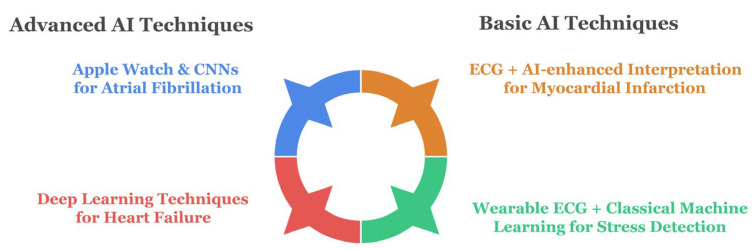
Summary of AI-ECG applications categorized by disease type and technological setting. ECG, electrocardiogram; AI, artificial intelligence; CNN, convolutional neural network. Each quadrant illustrates a clinical condition paired with a corresponding AI strategy and implementation context.

Figure 2 summarizes representative AI-ECG applications categorized by disease type and technological setting.

For a clearer overview, we organized the reviewed literature into two main clusters: (i) Clinical diagnostic applications based on cardiovascular conditions and (ii) integrated AI-ECG use in hardware-based monitoring systems. This section synthesizes these contributions, emphasizing their methodological approaches, model outputs, and clinical implications.

#### 3.1.1. Clinical Diagnostics by Disease Type

Several studies have focused on the deployment of AI models for detecting specific cardiovascular conditions using ECG data. These studies vary in terms of disease focus, training datasets, and AI methodologies employed. In the following subsections, we detail how AI has been applied to diagnose AF, HF, MI, and other arrhythmic or stress-related conditions. Each disease-specific synthesis highlights the performance benchmarks reported, common architectural choices (e.g., convolutional neural networks [CNNs], long short-term memory networks [LSTMs]), and the extent to which studies consider interpretability, bias, or generalization across populations.

##### Atrial Fibrillation Detection

AF is a major contributor to stroke and cardiovascular mortality worldwide. The combination of wearable technologies and DL has enabled novel paradigms for early detection and personalized risk prediction of AF. Meta-analyses report pooled sensitivities and specificities exceeding 94% for Apple Watch–based AF detection, though heterogeneity across device versions and study designs remains a concern [7,24].

**Figure 3:**
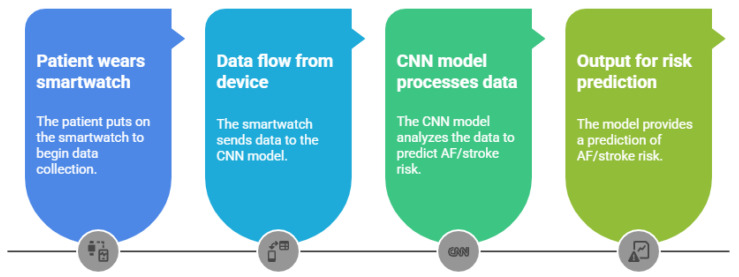
Wearable-AI pipeline for AF detection. AI, artificial intelligence; AF, atrial fibrillation; CNN, convolutional neural network.

Figure 3 illustrates a representative end-to-end pipeline for wearable AI–based AF detection and stroke risk prediction [25]. A patient wears a smartwatch that continuously captures single-lead ECG data. These data are transmitted to a CNN model, either cloud-based or local, which processes the signals to detect AF episodes. The output confirms the presence of AF and can also be integrated with clinical variables to assess the patient’s long-term risk of stroke.

Recent large-scale studies—particularly the meta-analysis by Shahid et al. [7]—have demonstrated the clinical potential of wearable AI–based AF detection. Shahid et al. reported a pooled sensitivity of 94.8% (95% confidence interval (CI): 91.7–96.8%) and a specificity of 95% (95% CI: 88.6–97.8%) for Apple Watch ECG detection of AF [7]. Other systematic reviews have found that wearable ECG devices achieve high accuracy in older adult cohorts, though the performance might vary by device and algorithm [11]. Some CNN-based models trained on datasets with multi-million records have shown excellent diagnostic performance (e.g., specificity > 99%), but evidence of prediction of incident AF years before onset remains limited and has not been clearly documented in the existing literature [15].

Despite promising results, several limitations remain. Device variability, lack of interoperability, and privacy concerns hinder the integration of wearable-AI systems with electronic health records (EHRs) [2,13]. Additionally, generalizability continues to be a major concern, as many AI models are trained on relatively homogeneous populations, limiting their applicability to diverse clinical settings. This issue has been emphasized in applied studies [2] and in methodological reviews highlighting the risks of biased training data and overfitting in neural network models [26].

The need for external validation and real-world testing, particularly in underrepresented demographic groups, has been increasingly recognized across the literature [2,26]. Ongoing efforts aim to overcome these barriers by standardizing ECG data formats, integrating AI pipelines into telemedicine platforms, and improving model explainability to enhance clinical trust and usability [27,28]. Reviews on wearable ECG monitoring reinforce the clinical promise and implementation challenges of these systems [28,29].

##### Myocardial Infarction Identification

The diagnosis of acute MI has traditionally relied on the presence of ST-segment elevation on ECG data. However, emerging evidence indicates that up to 56% of occlusion MIs (OMIs) may present without ST elevation on ECG, leading to missed or delayed diagnoses and poor clinical outcomes [4]. This realization has fueled a shift toward occlusion-based paradigms, where the presence of a coronary artery occlusion, rather than ST elevation on ECG alone, drives the diagnosis of MI and urgency of treatment.

AI is playing a central role in this paradigm shift by enabling the identification of subtle and non-ST-based ECG features and by supporting the integration of multimodal patient data—including ECG signals, imaging findings (e.g., echocardiography, computed tomography, etc.), and clinical variables—for improved ischemia detection [30,31].

**Figure 4:**
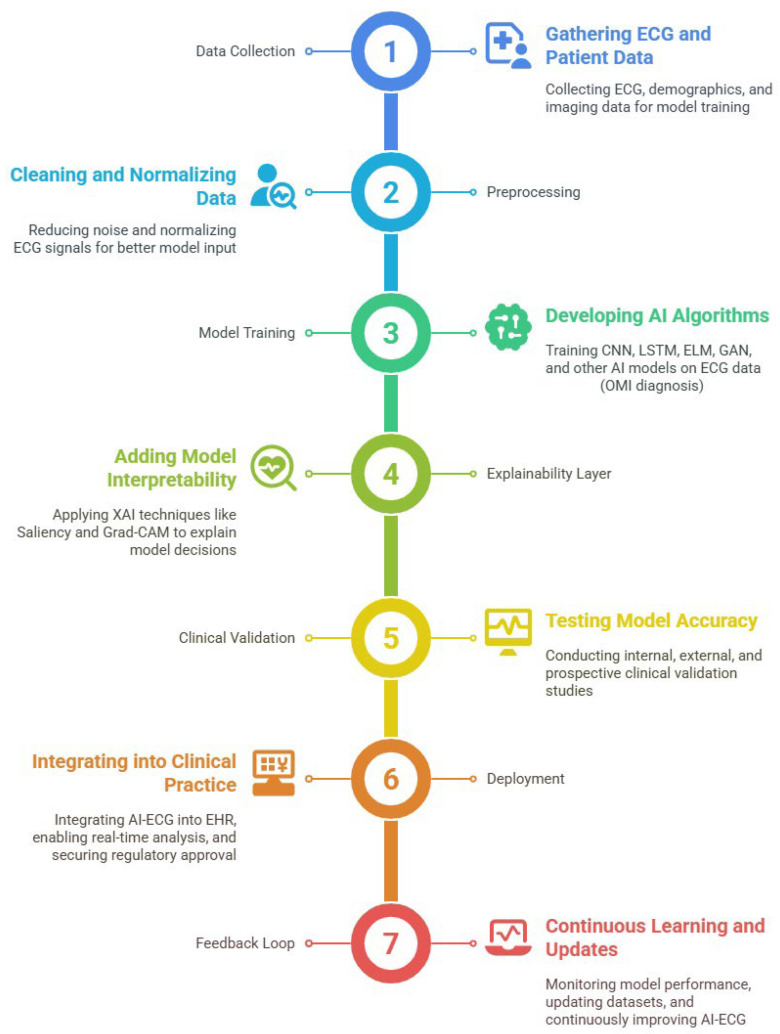
Multimodal fusion for ischemia diagnosis. ECG, electrocardiogram; AI, artificial intelligence; CNN, convolutional neural network; LSTM, long short-term memory networks; ELM, extreme learning machine; GAN, generative adversarial network; OMI, occlusion myocardial infarction; Grad-CAM, gradient-weighted class activation mapping; XAI, explainable AI; HER, electronic history record.

Figure 4 illustrates this multimodal fusion process, illustrating how an AI model can unify ECG data, imaging results, and clinical history to improve diagnostic accuracy for OMI. Studies by Xiao et al. [30] and Zhu et al. [31] exemplify these architectures, demonstrating that combining signal-based and image-based data in DL frameworks can yield superior performance compared to single-modality models.

The real-world implementation of multimodal fusion processes remains challenging due to the need for fast, automated interpretation and seamless integration into emergency response workflows. The “Código OMI” initiative in Spain serves as a case study, where AI-assisted triage tools are being deployed to reduce time-to-reperfusion in suspected OMI cases [4]. Nevertheless, studies such as Singh et al. [32] emphasize that standardized clinical protocols and large-scale prospective validation are still required to fully establish the safety and efficacy of AI-augmented decision-making.

Recent studies have highlighted various AI methodologies beyond traditional rule-based systems. For instance:

- Huérfano-Maldonado et al. [18] identified CNN architectures in multimodal settings.
- Yu et al. [9] proposed a “subtraction ECG” approach for dynamic serial analysis, showing that this method improves early diagnosis compared with static ECG evaluation.
- Gautam et al. [29] explored context-independent ML models that can detect MI even in atypical presentations, highlighting their potential in generalized deployment, though their study focused broadly on HF and remote monitoring.

These insights are summarized in Table 1, which compares rule-based, CNN, multimodal fusion, dynamic ECG, and context-free models. The findings reinforce that AI-driven approaches outperform conventional ST-segment elevation MI paradigms, particularly in the identification of subtle or atypical ischemic events.

In addition to the studies in Table 1, a growing body of evidence supports the efficacy of AI in detecting early and atypical MI. For instance:

- Elvas et al. [33] demonstrated improved sensitivity for non-ST elevation infarctions by integrating 12-lead ECG with echocardiographic features.
- Sun et al. [34] applied ensemble learning techniques to prehospital ECGs, enabling earlier ischemia recognition in out-of-hospital settings.
- Tao et al. [35] used magnetocardiography and AI to detect ischemia and coronary stenosis in patients without classical ECG changes.
- Finally, Lefebvre and Hoekstra [36] emphasized the need for next-generation ECG techniques, such as body surface mapping, to improve sensitivity in the detection of early MI.

Together, these studies illuminated a clear and consistent trend. AI-enhanced multimodal strategies, especially those integrating ECG, imaging, and clinical data, can improve the sensitivity, speed, and reliability of MI diagnosis. Therefore, such strategies are expected to inform future updates to emergency care protocols and clinical guidelines.

##### Heart Failure Monitoring

The use of AI for ECG-based detection of HF and left ventricular dysfunction represents one of the most transformative advances in contemporary cardiovascular diagnostics. Multiple large-scale studies have demonstrated that DL architectures, such as DenseNet-121, CNN-LSTM, and CNN-BiLSTM, can extract nuanced features from ECGs that are imperceptible to human readers, enabling the detection of reduced left ventricular ejection fraction (LVEF) with remarkable accuracy [1,18,37,38,39].

For instance, Hou et al. [1] trained a DenseNet-121 model on more than 136,000 paired ECG–echocardiogram datasets, achieving an AUC of 0.965 in internal validation and 0.848 in external testing, significantly outperforming traditional rule-based methods. These results underscore the diagnostic value of AI-enhanced ECG analysis in detecting subclinical ventricular dysfunction. Furthermore, Lee et al. [2] demonstrated that an ECG-based AI model could predict one-year mortality in patients with HF, offering a powerful tool for risk stratification and longitudinal care.

The predictive utility of ECG-based markers extends beyond structural dysfunction. Resting heart rate and heart rate variability, as captured in standard ECGs, have shown prognostic significance across large HF cohorts [40,41]. These findings reinforced the potential of AI to convert routine ECGs into dynamic, high-yield diagnostic and prognostic instruments.

To enhance generalizability, some studies have employed large, multiethnic datasets, such as the Clinical Outcomes in Digital Electrocardiography and Medical Information Mart for Intensive Care-IV repositories [42], which provide a broader representation of patient demographics. In contrast, single-center studies, such as the one by Attia et al. [39], often report excellent internal performance but might overestimate real-world applicability due to limited population heterogeneity [2,42]. This finding highlighted the ongoing need for external validation and prospective multicenter trials.

Several recent studies have aimed to address issues of model reliability and uncertainty. In particular, CNN-BiLSTM models designed by Seoni et al. [37] and Cheng et al. [38] incorporated uncertainty estimation, enabling them to provide CIs for their predictions—an essential feature for clinical adoption and decision support.

Beyond conventional DL, ELMs, and their variants have been investigated for their computational efficiency and competitive performance, especially in low-resource settings. Reviews by Wang et al. [17] and Huérfano-Maldonado et al. [18] highlighted that ELM-based models require minimal training time while offering acceptable accuracy. Furthermore, the use of random-weight neural networks in a previous study provided an additional methodological perspective for rapid ECG analysis [26].

Despite these advances, HF monitoring using AI-based methods faces some challenges. The lack of demographic diversity in training datasets remains a major limitation, contributing to algorithmic bias and diminished performance in underrepresented populations [2,7]. Additionally, the retrospective nature of most published studies, along with the scarcity of prospective, multicenter clinical trials, continues to hinder broad clinical translation [42,43].

Table 2 offers a comparative overview of some recent studies on AI-based detection of HF and reduced LVEF using ECG data. It presents key model characteristics, dataset scale and type, and performance metrics for internal and external validation. This synthesis highlighted the promising yet variable results achieved across different model architectures and study designs.

Table 2 reveals a consistent pattern of high internal AUC values (>0.92), underscoring strong predictive capabilities within controlled datasets. However, the external AUC values were notably lower and more variable, reflecting challenges in generalization and highlighting the importance of external testing. The inclusion of uncertainty estimation in some models adds interpretability and robustness; however, demographic bias and dataset limitations remain persistent obstacles.

Collectively, these findings reflected a growing consensus that the application of DL to ECG data offers a scalable, non-invasive, and effective solution for the early detection of cardiac dysfunction [40,43]. However, broader data inclusion, fairness-aware modeling, and prospective real-world validation are essential next steps to ensure equitable and clinically relevant AI deployment in HF diagnostics [41].

##### Stress and Anomaly Detection

ECG signals are highly susceptible to noise from muscle artifacts, baseline wander, and electrode motion, particularly in ambulatory and wearable contexts [8,9,44]. AI-based denoising techniques have emerged as superior alternatives to traditional filters. For instance, the dual-path interactive denoising autoencoder integrates CNN for local feature extraction and BiLSTM for temporal modeling, achieving substantial improvements in SNR and morphological preservation [44]. Wavelet thresholding combined with autocorrelation has also shown promise, especially in challenging noisy scenarios typical of wearable ECGs [9]. These methods enable accurate downstream classification, as demonstrated in studies using the MIT-BIH and PTB-XL datasets [9,44].

**Figure 5:**
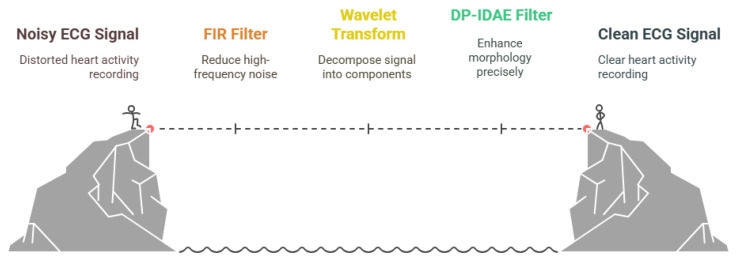
Comparison of denoising methods. ECG, electrocardiogram; FIR, finite impulse response; DP-IDEA, dual-path interactive denoising autoencoder.

Figure 5 provides a visual comparison of various denoising methods applied to ECG signals. The panel displays representative ECG traces at stages: the original clean signal, the same signal corrupted by typical noise artifacts, and the outputs after processing with three filtering techniques, namely finite impulse response filtering, wavelet thresholding, and the advanced dual-path interactive denoising autoencoder (DP-IDAE). By visually juxtaposing these results, the figure highlights the superior ability of state-of-the-art AI-based denoising approaches to preserve diagnostic morphology and restore signal quality, compared to traditional filtering methods. This juxtaposition helps underscore the importance of advanced signal processing for ensuring accurate downstream ECG interpretation in clinical and wearable device settings.

Recent reviews highlighted the need for standardized benchmarks and open datasets to compare denoising algorithms and facilitate reproducibility [12]. Furthermore, real-time implementation and computational efficiency remain active areas of research [17,18]. Table 3 provides a comprehensive comparison of advanced signal processing methods applied to ECG data, focusing on their effectiveness in noise reduction and preservation of clinically relevant features.

This table summarizes the performance of traditional filtering techniques and state-of-the-art AI-based approaches, such as DP-IDAE and novel wavelet thresholding methods. Key metrics, including improvements in SNR, root-mean-square error, and morphological similarity, are presented alongside the datasets used for evaluation. By highlighting the regularities and outcomes across multiple studies, the table foregrounds the growing consensus that AI-driven denoising techniques outperform conventional filters while preserving the diagnostic integrity of ECG signals, which is essential for accurate downstream classification and clinical interpretation.

The findings summarized in Table 3 have been corroborated by numerous other research studies. Petmezas et al. provided a state-of-the-art overview of DL approaches for ECG denoising [45], and an earlier survey by Mosslah et al. outlined classical noise-removal techniques and challenges [46]. Researchers have proposed real-time denoising autoencoders for wearable devices [47,48] and demonstrated noise-robust DL architectures that maintain classification accuracy under heavy noise [49,50].

Comparative studies and systematic evaluations [51,52,53,54] have consistently shown that modern AI models can significantly improve signal quality (often achieving SNR gains) while preserving clinically important waveform features. Collectively, these studies reinforced that advanced filtering and ML methods are crucial for reliable ECG analysis in noisy real-world environments. Standardized evaluation frameworks for these algorithms are still needed to fairly compare performance across studies [12,45,46,55]. However, the trend clearly favors AI-enhanced signal processing for clinical and consumer ECG applications.

#### 3.1.2. Explainable AI for ECG Diagnosis

The “black box” nature of many DL models remains a major barrier to clinical trust and adoption. Recent studies have applied post hoc XAI methods, such as saliency maps, gradient-weighted class activation mapping (Grad-CAM), and integrated gradients, to CNN models, leading to the identification of clinically relevant ECG segments and facilitating models [56,57]. For example, Kapsecker et al. [13] and Ayano et al. [15] demonstrated that saliency-based heatmaps over ECG waveforms correspond to known diagnostic features, helping clinicians understand why the AI made a particular prediction [58].

**Figure 6:**
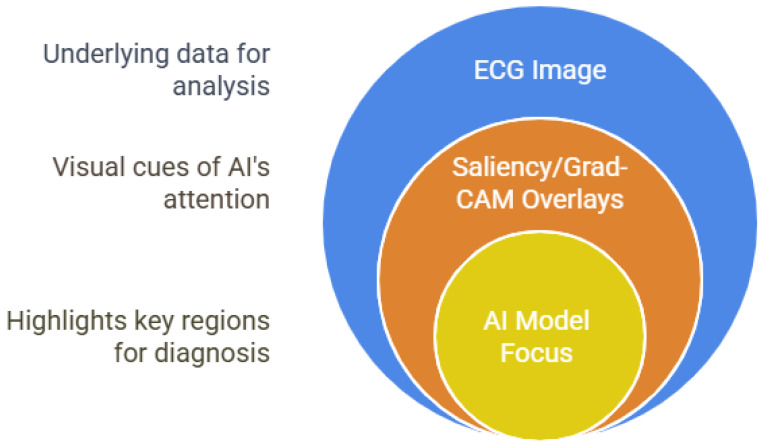
XAI heatmap on ECG. XAI, explainable artificial intelligence; ECG, electrocardiogram; Grad-CAM, Gradient-weighted Class Activation Mapping; AI, artificial intelligence.

Figure 6 visually demonstrates how XAI techniques can be applied to ECG analysis. It shows an ECG trace overlaid with color-coded heatmaps, highlighting the specific regions and waveform segments that the AI model considers most influential for its diagnostic decision. By making the model’s focus transparent, these visualizations help clinicians understand, validate, and trust the AI’s predictions, bridging the gap between complex DL algorithms and clinical interpretability.

This approach supports model auditing and regulatory compliance and also opens new avenues for discovering novel ECG biomarkers and improving the overall reliability of AI-assisted cardiac diagnostics.

Systematic reviews have also revealed significant inequities in ECG datasets: only 29% of the datasets reported patient race/ethnicity, 89% of the recordings were derived from White/Caucasian individuals, and just 28% of the datasets were publicly accessible [4,12]. This bias threatens model generalizability and fairness, as highlighted in previous studies [2,13]. Actionable recommendations include mandating diversity reporting, standardizing metadata, and promoting public dataset availability [2,12]. The integration of XAI tools in clinical workflows is also essential for transparency and regulatory compliance [6].

Table 4 summarizes the principal findings and actionable recommendations from recent studies addressing explainability and fairness in AI-based ECG analysis [59]. The table highlights consensus in the literature regarding the importance of integrating XAI tools, such as saliency maps and Grad-CAM, to enhance transparency and clinical trust in model predictions. In the table, key issues are paired with consensus observations and recommendations.

The information synthesized in Table 4 also draws attention to persistent challenges, including demographic bias, under-representation of minority groups in training datasets, and limited public accessibility of ECG data. By juxtaposing these issues with proposed solutions, such as mandatory diversity reporting, standardized metadata, and increased availability of open-access datasets, the table provides a concise roadmap for advancing the interpretability and equity of AI-ECG technologies in clinical practice.

Beyond the specific examples cited in Table 4, a growing body of literature has been devoted to improving explainability and fairness. Multiple researchers have proposed and reviewed techniques to interpret ECG model decisions, ranging from rule-based surrogate models to novel visualization methods [44,60,61,62,63].

These studies have consistently reported that introducing explainability (e.g., through feature attribution or case-based reasoning) enhances clinicians’ acceptance of AI outputs. In parallel, concerns about algorithmic bias have prompted investigations into bias mitigation strategies, such as re-sampling, adversarial debiasing, and federated learning, to ensure that AI benefits are equitably distributed [64,65,66,67,68].

Comprehensive surveys of interpretability and fairness in AI-ECG applications [64,65,69,70,71] have echoed the consensus that technical advances must be paired with transparency and rigorous bias audits before AI-ECG models can be widely trusted. Taken together, these efforts signal a maturation of the field; the community is moving beyond pure performance metrics to addressing how and for whom these AI systems work.

### 3.2. Emerging Applications: Stress, Real-Life Monitoring, and Anomaly Detection

The integration of wearable technologies into daily life has created novel opportunities for real-time physiological monitoring, enabling AI-driven detection of stress and other abnormal health events. Among these, stress detection and anomaly detection models have gained traction as tools for early identification of arrhythmias, ischemic events, and physiological instability in free-living conditions.

#### 3.2.1. Stress Detection in Daily Life Using Wearable ECG

**Figure 7:**
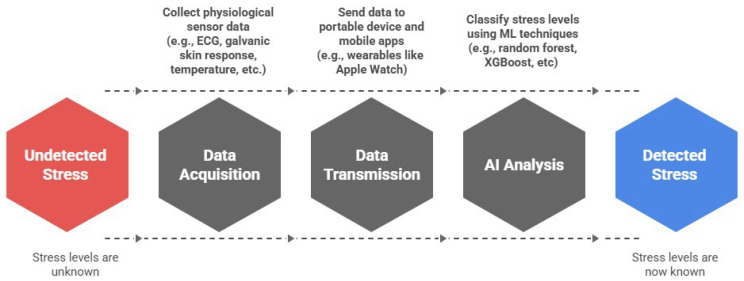
Real-time stress detection system. ECG, electrocardiogram; ML, machine learning; XGBoost, extreme gradient boosting.

Stress is a key risk factor for CVD. Therefore, detecting stress using wearable technologies is now a critical target for continuous monitoring in preventive cardiology and mental health applications. Figure 7 presents a conceptual architecture for a wearable ecosystem designed for real-time stress detection. This model includes a network of physiological sensors (e.g., ECG, galvanic skin response, temperature, etc.) that transmit data to portable devices, where ML algorithms such as random forest (RF) and extreme gradient boosting (XGBoost) analyze the signals to classify stress levels. This flow reflects an emerging paradigm that combines wearable sensing, mobile computing, and AI-driven analytics to enable continuous, passive monitoring in naturalistic settings.

Recent empirical studies support the feasibility and effectiveness of such systems. Abd Al-Alim et al. [11] demonstrated that RF and XGBoost models trained on the SWEET dataset achieved over 98% accuracy for binary and multi-level stress classification in free-living conditions. Hota and Park [72] reported that physiological signal–based ML methods performed well in stress detection tasks in practical settings. In a comprehensive systematic review, Pinge et al. [73] consolidated evidence from multiple wearable-based studies, confirming that multimodal sensing, combining ECG, skin conductance, and temperature, can significantly enhance the accuracy of stress classification. Finally, Canali et al. [74] highlighted broader implementation challenges for AI-based wearables, including concerns around data quality, interoperability, and algorithmic fairness—key considerations for deploying stress monitoring at scale.

Recent work on real-time anomaly detection using AI has leveraged DL architectures, including autoencoders and variational models, to capture subtle deviations in ECG morphology. Kapsecker et al. [13] employed a variational autoencoder (VAE) for unsupervised detection of anomalies in single-lead ECG signals, demonstrating the model’s capability to differentiate between normal and pathological signals without labeled training data. This development represented a critical step forward from earlier rule-based or supervised classifiers, which often required extensive annotation. Similarly, Smets et al. [75] used large-scale wearable data to extract digital stress phenotypes, linking outlier patterns to stress-induced physiological shifts, further reinforcing the overlap between stress monitoring and anomaly detection.

Compared to earlier approaches, which were often limited to single-signal analysis or laboratory settings, these recent studies have achieved meaningful progress by:

- Utilizing multimodal sensor fusion, combining ECG with galvanic skin response, temperature, and motion data for more robust detection;
- Validating models in ecologically valid, real-world contexts, rather than artificial experimental setups; and
- Adopting advanced ML models that outperform traditional statistical techniques in terms of sensitivity and generalizability.

These advances are consistent with broader reviews highlighting the technical viability and increasing clinical relevance of AI-driven wearable systems for stress detection [29,74].

However, the implementation of wearable systems for stress detection still faces some challenges. Long-term deployment must address potential model drift, signal noise in ambulatory conditions, and personalization across diverse populations. To bridge these gaps, future research should:

- Promote standardized, multiethnic datasets to evaluate generalizability across global populations [74];
- Investigate adaptive learning models capable of updating in response to user feedback or contextual changes; and
- Develop consensus on validation protocols and clinical benchmarks, such as those established in cardiovascular AI research.

Wearable AI systems for stress detection are transitioning from proof-of-concept to scalable clinical tools. The synergy between sensor technology and ML offers a promising pathway for integrating stress monitoring into holistic health and wellness strategies [76].

#### 3.2.2. Real-Time Anomaly Detection with Tailored Deep Learning Models

Personalized, real-time anomaly detection is emerging as a key functionality of wearable and bedside ECG monitoring. Traditional ML approaches, such as support vector machines, decision trees, and fixed-threshold algorithms, often suffer from high false positive rates in ambulatory settings, largely due to their inability to account for inter- and intra-individual variability in cardiac signals [77]. These models are typically trained on population-wide data and fail to adapt to patient-specific ECG baselines, making them suboptimal for continuous, context-aware monitoring.

To overcome these limitations, recent research has shifted focus toward deep generative networks, especially generative adversarial networks (GANs), and representation learning models, particularly VAEs and their variants. These architectures allow models to learn a subject’s physiological baseline and identify deviations without relying on manually labeled anomalies. While VAEs are instrumental in anomaly detection, GANs have also shown promise in this application. Mirza et al. [78] explored the use of skip connections in GAN architectures for improved signal synthesis. Though their work focused on medical image generation, the architectural innovations are directly applicable to ECG modeling, especially in tasks such as anomaly simulation, data augmentation, or training of robust detectors under rare-event conditions.

Nowroozilarki et al. [79] applied VAEs to morphological clustering and noise detection tasks, demonstrating that the model could distinguish noise artifacts from genuine physiological anomalies. Their results supported the hypothesis that personalized latent representations can improve the specificity and robustness of anomaly detection, particularly in noisy, real-life signal conditions.

Kapsecker et al. [13] employed a β-total correlation VAE to extract disentangled latent variables from single-lead ECGs, enabling unsupervised detection of clinically relevant deviations, such as ectopic beats or waveform irregularities.

Complementarily, Dhyani and Butola [77] comprehensively surveyed the use of autoencoders in ECG anomaly detection. They emphasized how encoder–decoder structures can reconstruct baseline ECG signals and flag unexpected deviations, a method that naturally fits into personalized, real-time workflows. Compared to classical classifiers, autoencoder-based models have been shown to adapt better to subtle, patient-specific changes.

From a broader ecosystem perspective, Smets et al. [75] leveraged wearable data at scale to uncover digital stress phenotypes, illustrating how unsupervised models can detect patterns in real-world settings that would be difficult to classify using static methods. Their approach underscored the importance of context-aware and temporally adaptive detection, where baseline drift and behavioral patterns can be modeled dynamically.

Moreover, Raje et al. [80] developed an online framework for anomaly detection using ML in healthcare Internet of Things environments. Their model supported real-time learning and detection, a critical feature for continuous ECG monitoring, particularly in telehealth and remote-care settings. Hong et al. [81] further reviewed the capabilities of DL in ECG analysis, emphasizing that advanced architectures such as autoencoders and one-class classifiers are outperforming traditional models in terms of sensitivity to rare, unexpected events.

Finally, Chukwu and Moreno-Sánchez [82] proposed an XAI model for 12-lead ECG–based arrhythmia detection, integrating personalized baselines into the model’s decision-making process. Their work bridged the gap between high-performing generative models and clinically interpretable outputs, a key step toward regulatory and bedside adoption.

The transition from generic classifiers to personalized, generative, and adaptive anomaly detection models marks a pivotal paradigm shift in cardiac monitoring. Compared to traditional techniques, these modern architectures—especially VAE-based, autoencoder-based, and attention-informed models—demonstrate greater accuracy, better handling of patient-specific variability, and superior integration into real-time wearable systems. Nevertheless, further research is needed to address challenges such as long-term model drift, dataset scarcity, and clinical validation, particularly in low-resource or ambulatory environments.

### 3.3. Synthetic ECG Generation with Deep Generative Models

Synthetic ECG data generation addresses the key challenges of data scarcity, class imbalance, and privacy in medical AI. Three main approaches have been identified in this context: mathematical modeling, traditional signal/image-based models, and DGMs, such as VAE, GAN, and diffusion models [12,83,84,85,86].

**Figure 8:**
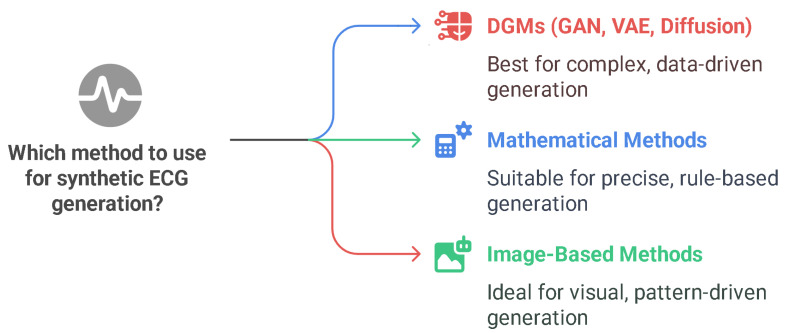
Taxonomy of synthetic ECG generation methods. ECG, electrocardiogram; DGMs, deep generative models; GAN, generative adversarial network; VAE, variational autoencoder.

This tripartite classification is illustrated in Figure 8, which summarizes the methodological landscape by grouping approaches based on their conceptual foundations and use cases: mathematical models prioritize rule-based waveform synthesis; image-based models favor pattern extraction through two-dimensional transformations; and DGMs offer high-capacity, data-driven synthesis tailored to complex clinical applications. This visualization helps identify methodological strengths and align generation techniques with specific research or deployment goals [87,88].

Synthetic ECG generation has become a critical research frontier at the intersection of cardiology and AI, addressing long-standing challenges such as data scarcity, class imbalance, and privacy preservation. As depicted in Figure 8, current methodologies for synthetic ECG generation are broadly categorized into three families: mathematical models, image-based methods, and deep generative models (DGMs), such as GANs, VAEs, and diffusion models [12,83,84]. Each paradigm contributes differently to the trade-off among realism, scalability, and clinical applicability.

- Deep Generative Models: Realism and Data-Driven Innovation

The most prominent advances have resulted from DGMs, especially GANs, VAEs, and diffusion models, which collectively aim to generate highly realistic and variable ECG signals while preserving clinical fidelity [12,83,86].

GANs are the most extensively explored DGMs. Goodfellow et al. [85], in their foundational work, introduced the adversarial learning paradigm that has since revolutionized synthetic biomedical signal generation. Thambawita et al. [89] developed a DeepFake ECG system using GANs that produced realistic 12-lead ECGs verified against a commercial ECG interpretation engine, reporting near-human indistinguishability. Similarly, Rayavarapu et al. [86] synthesized pathological ECG patterns that improved the performance of downstream arrhythmia classifiers by mitigating class imbalance, a result corroborated by Prakash and Subramanian [90] in a real-world classification setting.

However, GANs have their limitations. Mode collapse, instability during training, and difficulty in conditioning the output on specific labels have led researchers to explore alternatives and hybrid solutions [91,92,93].

VAEs, introduced in this domain by Adib et al. [83] and elaborated by Ibrahim et al. [93], offer probabilistic modeling of latent spaces, which enables controlled generation with fewer risks of overfitting. Ibrahim’s hybrid GAN-VAE framework captured global rhythm morphology and inter-patient variation, showing promise in generating rare waveform types and tailoring synthetic data to specific clinical needs. Upreti [94] extended this idea by implementing conditional VAEs for label-aligned ECG synthesis, improving control over the generated signal class.

Diffusion models, a more recent addition to the field, are based on reverse stochastic processes that iteratively denoise random noise to produce high-fidelity outputs. Heng et al. [92] and Ibrahim et al. [93] highlighted the superiority of diffusion-based synthesis in preserving morphological detail, especially for long sequences. Although computationally intensive, diffusion models offer a promising direction for future ECG synthesis, particularly when combined with transformers or conditional encoding.

- Mathematical and Image-Based Approaches

As discussed by Adib et al. [83], mathematical models, such as the McSharry or dynamical models, enable the generation of rule-based waveforms derived from physiological equations. These models offer high interpretability and parameter control but are limited by their rigidity and inability to capture the complex stochasticity of human ECG signals. Rahman et al. [88] reported that while these approaches are historically important, their use is now largely relegated to simulating idealized or baseline signals rather than pathological variations.

Image-based methods, often employing transformations of one-dimensional ECG signals into two-dimensional spectrograms or grayscale images, have gained attention due to the success of CNNs in image synthesis. Núñez et al. [84] demonstrated the utility of such representations for transfer learning and data augmentation in arrhythmia classification. However, Musa et al. [95] and Akpinar et al. [87] argued that these methods introduce artifacts and lose critical temporal dependencies, limiting their realism and diagnostic value.

- Comparative Evaluation and Validation Metrics

With the rapid proliferation of multiple generative models, the need for quantitative evaluation metrics has become paramount. Wang and Zhao (as cited by Adib et al. [83]) proposed using morphology similarity indices, heart rate variability measures, and spectral fidelity metrics as objective benchmarks. Akpinar et al. [87] recommended the use of beat-wise dynamic time warping scores, while Musa et al. [95] advocated for cross-domain classification performance as a surrogate indicator of synthetic data quality.

In their comprehensive meta-analysis on DL applications in ECG, Musa et al. [95] highlighted the risks of relying solely on visual inspection or basic waveform metrics to assess synthetic signal fidelity. They emphasized the necessity of incorporating clinical validation criteria, such as arrhythmia interpretability and diagnostic utility, to avoid overestimating the usefulness of synthetic data for real-world deployment. Raghu et al. [96] and Khamparia and Gupta [97] echoed this concern, calling for the development of standardized ECG challenge benchmarks, akin to ImageNet in computer vision, to benchmark synthetic ECG generators under consistent, replicable protocols.

- Synthetic ECGs for Privacy Preservation and Regulatory Compliance

Beyond data augmentation, synthetic ECGs offer a solution to privacy and data-sharing barriers in biomedical research. As highlighted by Goodfellow et al. [85] and further explored by Rahman et al. [88], synthetic ECGs, if statistically representative but unlinkable to real identities, can be shared across institutions without violating patient confidentiality. Thambawita et al. [89] labeled this possibility as “the beginning of the end for privacy concerns” in medicine. Ibrahim et al. [93] demonstrated synthetic ECG generation across multi-institutional settings using federated learning, enhancing generalizability while preserving local data privacy.

This capability is particularly relevant for global health research, where data governance frameworks vary significantly. The synthesis of de-identified yet realistic ECGs enables collaborations between low- and high-resource settings, such as in the study by Núñez et al. [84], which focused on arrhythmia datasets in Latin American populations.

- Technical Advancements and Tailored Synthesis

The field is also seeing innovations aimed at conditioning and customizing the generation process. Berger et al. [91] proposed conditional GANs that generate specific arrhythmia types on demand, whereas Ma et al. [98] combined multiple generative strategies to synthesize challenging waveform morphologies with improved inter-class separability. These innovations aligned with recent efforts by Ibrahim et al. [93] and Musa et al. [95] to create modular architectures capable of transfer learning and adaptation across multiple cardiac conditions.

Moreover, Dhyani and Butola [77] emphasized that autoencoders, including VAEs, outperform traditional augmentation methods in capturing fine-grained beat dynamics, particularly in unsupervised or low-data environments.

- Current Gaps and Future Directions

Despite rapid advances, multiple challenges remain in the domain of synthetic ECG generation. As noted by Escrivaes et al. [99], current models still struggle with edge-case morphologies, such as Brugada syndrome or rare conduction abnormalities. Moreover, very few models have been prospectively validated in clinical pipelines, a gap that is contextualized in this review by comparing validation strategies across studies (see Discussion section).

Finally, the field would benefit from open-access benchmark competitions and regulated synthetic ECG repositories, as proposed by Khamparia and Gupta [97] and further reinforced by Upreti [94] and Raghu et al. [96]. These efforts are essential to accelerate clinical integration and regulatory approval of AI systems trained on synthetic ECGs.

### 3.4. Clinical Validation and Implementation Challenges

#### 3.4.1. Generalizability and Bias

One of the most formidable obstacles to the clinical translation of AI-based ECG models is their limited generalizability, largely due to dataset bias, homogeneous population samples, and insufficient subgroup reporting. These issues have been extensively documented across the literature and raise pressing concerns about the equity and safety of AI tools in real-world clinical settings [2,4,7,12].

In their comprehensive mortality prediction study using AI-ECG, Lee et al. [2] found that less than one-third of ECG-based publications reported patient ethnicity, while most datasets originated from high-income countries in North America and Europe. This geographical bias, coupled with the under-representation of minority groups, led to models that perform well in the development stage but poorly in the deployment stage, particularly for non-White populations or patients with atypical ECG morphologies.

In a scoping review of synthetic ECG generation, Zanchi et al. [12] reinforced this concern by highlighting that most public datasets used to train AI-ECG models lack demographic metadata, such as race, sex, or age distributions. They argued that without such information, fair audits and stratified performance evaluations become nearly impossible, putting clinical reliability at risk.

These concerns were echoed in the policy-oriented review by McLaren et al. [4], who advocated for a paradigm shift in acute MI diagnostics, emphasizing that failure to validate AI models across diverse subpopulations might lead to misdiagnosis and delayed interventions, particularly in underserved groups.

Moreover, a meta-analysis by Shahid et al. [7] demonstrated variable diagnostic performance of wearable ECG devices such as the Apple Watch across demographic lines, serving as a warning that models tuned on non-representative data might amplify disparities rather than mitigate them.

To empirically address this issue, Ribeiro et al. [42] proposed a decentralized strategy by training a deep neural network using ECG data from multiple Brazilian hospitals. Their approach was not a formal federated learning (FL) setup; however, it preserved local data privacy while exposing the model to more diverse patient populations, improving generalizability without data sharing.

Crucially, Bayona et al. [67] explicitly recommended FL as a solution to this problem of generalizability. They described FL as an emerging architecture that enables cross-institutional training while retaining data locally, thus simultaneously enhancing privacy and diversity. By integrating demographic heterogeneity into the model training pipeline, FL holds promise for building more equitable AI systems, particularly in cardiovascular applications.

In parallel, Islam et al. [64] advocated for mandatory dataset documentation standards, including transparent disclosure of age, sex, and ethnicity composition, as well as subgroup-specific model performance. They noted that many high-performing AI-ECG models omit these key metrics, making it difficult to assess whether diagnostic gains are uniformly distributed or concentrated in privileged demographics.

On the methodological side, Salih et al. [66] emphasized the need to couple technical fairness strategies, such as adversarial debiasing or subgroup reweighting, with XAI frameworks that allow clinicians to interrogate whether predictions vary by subgroup. However, they cautioned that post hoc explanations without fair training data pose the risk of legitimizing biased outcomes, underscoring that transparency is not a substitute for equity by design.

Collectively, these findings revealed a consensus across the field: robust generalizability cannot be assumed; it must be engineered. This goal requires a multipronged approach that includes:

- FL for training across diverse, siloed datasets [67];
- Open-access multiethnic ECG repositories [42];
- Transparent demographic reporting standards [12,64]; and
- Fairness-aware model evaluation protocols [66,67].

Without these measures, the promise of AI to democratize cardiological care faces the risks of backfiring, entrenching, and even amplifying existing inequities rather than resolving them. The development of AI-ECG models must therefore proceed with deliberate attention to inclusion, representativeness, and regulatory accountability.

#### 3.4.2. Infrastructure and Usability

Despite the growing accuracy and clinical potential of AI-ECG models, their real-world usability and integration remain major bottlenecks. Literature has consistently reiterated that achieving practical impact requires addressing infrastructural compatibility, user interface design, real-time operability, and clinician trust, all within complex and diverse healthcare environments [64,100,101].

One of the core infrastructural challenges is the seamless integration of AI algorithms into existing EHR systems and clinical workflows. In their systematic review of AF detection using wearable ECGs, Shahid et al. [7] emphasized the importance of embedding AI functionalities into hospital telemetry systems without disrupting established protocols. Their findings resonated with those of Gautam et al. [29], who reported that current AI implementations for HF monitoring using wearables are still limited by fragmented health data ecosystems, latency in data transmission, and a lack of real-time feedback mechanisms. Both studies converged on the need for standardized interfaces and application programming interfaces that allow interoperability between bedside devices, ECG carts, and backend AI engines.

Furthermore, usability hinges not only on technical feasibility but also on clinician interaction and trust. Islam et al. [64] highlighted that healthcare providers tend to place more trust in clinical adoption models that provide interpretability, such as by identifying the ECG leads that contributed most to a diagnosis. This finding was echoed by Zhou and Tan [102], who reported that their hybrid DL architecture with an embedded “explanation module” elicited enhanced clinician confidence and improved feedback rates.

However, user trust is fragile. Kelly et al. [100] argued that many physicians harbor skepticism toward AI predictions due to limited algorithmic literacy and insufficient training, an issue corroborated by Canali et al. [74], who showed that usability and acceptance increase when end-users are involved early in the design loop. These authors advocated for iterative, human-centered design models where clinicians can provide feedback and override AI outputs when necessary. In the domain of stress detection using wearables, Smets et al. [75] similarly found that user-centric iterations significantly enhance adoption, especially when AI outputs are tailored to clinical language and format expectations.

Latency and overload are other critical concerns. Multiple sources have stated that AI-ECG systems can generate high volumes of alerts, many of which are false positives or redundant, leading to alert fatigue [74,100,101]. To counter this, Smets et al. [75] and Natarajan and Subramanian [90] have recommended the implementation of intelligent triaging systems—algorithms that adaptively filter and prioritize alerts on the basis of signal quality, context, and historical data.

In terms of technical innovation, edge computing is emerging as a promising avenue. Gautam et al. [29] suggested deploying lightweight AI models on wearable devices or local hospital servers to reduce latency and ensure data privacy. This finding aligned with recommendations from Canali et al. [74], who also stressed the importance of interoperability across devices, emphasizing that systems must accommodate hospital-grade and patient-generated data from consumer wearables.

Infrastructural readiness extends beyond hospitals. In ambulatory and remote care settings, real-time ECG analysis from wearables must interact with mobile apps, cloud platforms, and patient portals. The challenges here are not only bandwidth and power efficiency but also security and regulatory compliance [74]. Implementing these systems requires close collaboration among developers, hospital information technology staff, clinicians, and regulatory experts to ensure that AI-ECG systems are clinically meaningful and ethically sound [100,101].

Infrastructure and usability form a crucial axis in the translational path of AI-ECG tools. Studies concur on the opinion that technical performance alone is insufficient—deployment success depends on trust, transparency, interoperability, and clinician engagement. As such, the field must continue to evolve from proof-of-concept AI models to robust, user-validated, and regulation-compliant clinical solutions. The literature [7,29,64,74,75,90,100,101,102] provides encouraging case studies and frameworks, but widespread adoption will demand sustained interdisciplinary efforts and shared guidelines for implementation in diverse clinical contexts.

#### 3.4.3. Regulatory Frameworks

**Figure 9:**
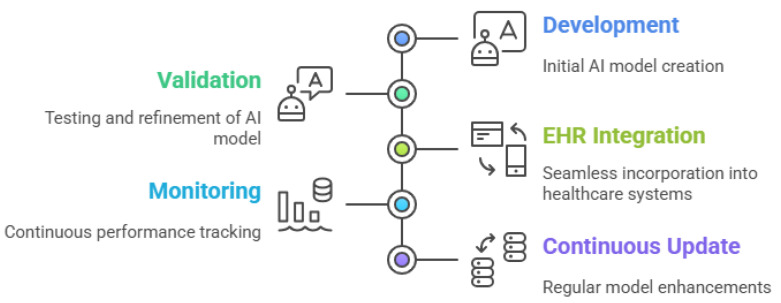
AI-ECG lifecycle in clinical practice. AI, artificial intelligence; ECG, electrocardiogram; EHR, electronic health record.

Despite the growing technical maturity of AI-ECG systems, regulatory frameworks remain one of the most formidable barriers to their clinical deployment. The lifecycle of AI in ECG analysis spans the stages of development, validation, EHR integration, monitoring, and continuous update, all of which introduce distinct regulatory demands (Figure 9). Regulatory bodies such as the Food and Drug Administration (FDA), European Medical Device Regulation, and Health Canada have begun to compile guidelines; however, their implementation is uneven, and harmonization remains limited across jurisdictions [100].

The figure outlines each critical phase, from initial model development and rigorous validation to seamless integration with EHRs, ongoing real-world monitoring, and continuous model updating based on new data and clinical feedback. This visual framework highlights the importance of robust technical performance and adaptability, regulatory compliance, and clinician engagement throughout the AI-ECG lifecycle. By capturing the full spectrum of development, deployment, and maintenance, the figure underscores the dynamic and evolving role of AI in delivering safe, effective, and sustainable cardiovascular care.

A central regulatory concern is the validation and reliability of AI predictions in real-world settings. Traditional medical devices undergo rigorous premarket evaluation, but AI systems, particularly those with learning capacity, challenge this paradigm. As Monfredi et al. [6] emphasized that deploying AI for detecting early clinical deterioration via continuous ECG monitoring mandates stringent oversight to ensure that the models generalize across dynamic, high-risk environments. This necessity was echoed by Shahid et al. [7], who underscored that even FDA-cleared wearables for AF detection, such as the Apple Watch, require postmarket surveillance and real-world performance evaluation to retain approval.

In response, regulatory initiatives such as the FDA’s Good Machine Learning Practice emphasize that, in addition to initial clinical validation, continuous performance monitoring and explainability are necessary throughout the product’s lifecycle [100]. This approach is reflected visually in Figure 9, where each phase, from development to update, involves ongoing regulatory scrutiny. Key to this vision is the establishment of “predetermined change control plans”: mechanisms that allow AI models to evolve within predefined safety bounds without undergoing complete reapproval. However, as of 2025, no AI-ECG model has received clearance under such adaptive paradigms [100].

Another critical issue is algorithmic transparency and explainability. Regulatory confidence increases when clinicians can understand, audit, and verify how an AI model reaches its conclusions. Ayano et al. and Islam et al. have argued that integrating interpretable ML methods, such as feature relevance highlighting or lead-wise attribution, can ease regulatory pathways and support clinician trust [15,64]. Furthermore, as Kelly et al. noted, regulators are increasingly requiring formal documentation of interpretability mechanisms and traceability as part of the submission process [100].

The use of synthetic data for development and validation has also introduced novel regulatory questions. Zanchi et al. and Goodfellow et al. pointed out that although synthetic ECGs generated via GANs offer advantages of data augmentation and privacy preservation, their use is not yet standardized under current validation frameworks [12,85]. Regulatory bodies have not yet outlined how synthetic data should be assessed for realism, bias, and physiological plausibility before being used in clinical-grade AI models.

Additionally, the integration of AI systems with hospital infrastructure presents regulatory and legal implications. Islam et al. and Shahid et al. have highlighted that embedding AI into EHRs, telemetry systems, or remote monitoring platforms requires safeguards for cybersecurity, system interoperability, and liability delineation, particularly when AI outputs are acted upon autonomously [7,64].

Furthermore, governance structures are underdeveloped. Kelly et al. stressed that interdisciplinary collaboration among clinicians, developers, and regulators is essential to align AI capabilities with clinical utility and regulatory expectations [100]. They recommended the creation of AI-specific Institutional Review Boards or algorithm oversight committees to provide ongoing assessment post-deployment, especially for continuous learning systems.

Regulatory clearance for AI-ECG tools involves more than merely proving predictive accuracy. It requires robust evidence of safety, fairness, explainability, security, and adaptability, all while navigating evolving regional and international standards. Figure 9 encapsulates this iterative regulatory process by highlighting the interconnected phases where governance must be embedded. Until regulatory science evolves to fully support adaptive, transparent AI models, achieving widespread clinical adoption of AI-ECG will remain an incremental, tightly supervised endeavor.

## 4. Discussion

The rapid advancement of AI in electrocardiography has produced a wealth of new evidence; however, contextualizing these findings within the broader landscape of published reviews is an essential task. Our synthesis showed that DL and ML models consistently outperform traditional ECG interpretation in diagnosing HF, acute MI, and AF [1,7,8]. This finding was in line with the findings from the comprehensive review by Kim et al. [15], showing that CNN and hybrid models routinely achieve higher sensitivity and specificity than rule-based or classical statistical methods, particularly in arrhythmia detection.

A notable point of convergence across multiple reviews was the transformative potential of AI in the early detection of left ventricular dysfunction. Hou et al. [1] demonstrated that DenseNet-121 and related architectures, when trained on large multicenter datasets, can achieve AUCs exceeding 0.95 for HF screening. These results echoed the findings from recent analyses of ELMs and random-weight networks [17,18,26,103,104], which highlighted the value of ELMs and their variants for rapid and robust ECG classification in biomedical signal processing.

However, as emphasized by Hou et al. [1] and Zanchi et al. [12], the generalizability of these models is often hampered by limited demographic diversity and insufficient external validation. Our review supports this concern. Only about 29% of the datasets reported race or ethnicity, and most were dominated by White/Caucasian individuals. This observation underscored the need for more inclusive data collection and reporting standards, a recommendation echoed by studies on dataset diversity [3,64,66].

In the context of MI, recent paradigm shifts, such as the move from ST-segment elevation MI/non-ST-segment elevation MI to OMI/non-OMI, are strongly supported by AI-driven research [4,76]. Our findings aligned with those of Ahmed and Sullivan, who showed that multimodal AI models integrating ECG, echocardiography, and imaging data outperform traditional ST-elevation criteria, especially in terms of detecting subtle or atypical presentations of ischemia [29]. These findings call for clinical protocols to be updated [4]. Notably, other studies have reported that AI-based models can help identify ischemia even in ECGs that appear normal by traditional criteria, reinforcing the OMI paradigm change [33,34].

Robust evidence supports the use of AI for predicting the risk of AF and stroke. Shahid et al. and Abd et al. reported that wearable devices, when combined with DL algorithms, achieve high sensitivity and specificity [7,11]. Cheng et al. [38] further demonstrated that CNNs trained on millions of ECGs can predict current and future risks of AF with high accuracy, a finding corroborated by Ayano et al. [15]. This convergence suggested that AI-enhanced screening for AF (for example, via consumer smartwatches) is approaching a level of reliability suitable for widespread use, although issues of false positives and clinical follow-up remain to be fully addressed [28,105].

Noise reduction and signal processing remain critical challenges. Our review found that dual-path autoencoders [83] and advanced wavelet thresholding [9] outperform traditional filters, a result corroborated by other recent reviews [12,18]. These methods are particularly important for wearable and ambulatory ECG, where signal quality is often compromised. The consensus across signal processing literature is that no single denoising technique fits all scenarios; thus, hybrid approaches and adaptive filters are gaining attention [45,46,52]. Recent literature has highlighted the emergence of synthetic ECG data generation using GANs, VAEs, and diffusion models [9,12].

While these approaches help address class imbalance and privacy concerns, researchers in the field agree that standards for clinical plausibility and validation of synthetic data are urgently needed [12,89]. Encouragingly, multiple research groups are now proposing evaluation benchmarks for synthetic ECGs and even holding competitions to improve realism [83,97].

Explainability and fairness are recurring themes. Monfredi et al. and Kapsecker found that XAI tools, such as saliency maps and Grad-CAM, enhance clinician trust and model auditability [6,13]. However, Lee et al. [2] and Zanchi et al. [12] pointed out that the lack of dataset diversity and transparency continues to limit the equitable deployment of AI-ECG models.

Our review indicated that addressing these issues is paramount for the next phase of AI integration into healthcare. An AI model that performs brilliantly on one population but poorly on another cannot be considered a success. Our synthesis is unique in comparison to other recent reviews [106,107,108,109] because, in addition to arrhythmia detection, it covers HF, ischemia, wearable applications, and advanced signal processing. This broader perspective reveals both the strengths and persistent gaps in the field. Table 5 offers a comparative overview of major international articles that have addressed the intersection of AI and ECG in recent years.

A deep analysis of Table 5 reveals that while numerous previous reviews have explored the intersection of AI and electrocardiography, the present review offers a substantively distinct contribution by addressing persistent gaps in methodological rigor, clinical applicability, and translational depth. Drawing on a comparative analysis of over 35 major reviews, this review distinguishes itself across ten key dimensions:

1. Systematic and Comprehensive Scope

Our review applied PRISMA methodology and synthesized over 100 peer-reviewed studies published between 2019 and 2025, surpassing most existing reviews in breadth and transparency. Only a few works (e.g. [25,27,64,67], etc.) report similar volume, and those reviews lack the multi-dimensional approach adopted herein.

2. Multi-Disease Coverage

Many reviews have focused on a single condition, such as AF, HF, or arrhythmia. In contrast, our review integrated findings across a wide spectrum of CVDs and related analytical tasks, including AF, HF, MI, ventricular tachycardia, and stress/anomaly detection, providing a holistic synthesis aligned with real-world clinical demands.

3. Comparative Model Performance

We offered a detailed comparative evaluation of the diagnostic efficacies of DL techniques such as CNNs, recurrent models such as LSTMs, and hybrid architectures. Such a comparison was often missing or only qualitative in nature in previous reviews.

4. XAI and Interpretability

Our synthesis evaluated model-specific explainability techniques (e.g., Grad-CAM, Shapley Additive Explanations, attention mechanisms, etc.) and discussed their diagnostic value—a domain often overlooked in previous reviews, which were mostly limited to algorithmic accuracy.

5. Synthetic ECG Generation

Unlike most reviews that mentioned synthetic signals in passing, we examined their role in addressing data scarcity, privacy concerns, and class imbalance in detail. We mapped generative methods (e.g., GANs, VAEs, etc.) to clinical use cases and validation challenges.

6. Wearables and Real-World Signal Variability

Our review explicitly incorporated studies on wearable ECG acquisition, analyzing how motion artifacts and sensor placement affect AI performance and generalizability—elements under-represented in most previous surveys.

7. Noise Reduction and Signal Preprocessing

One of the most critical and overlooked areas in the literature was ECG signal noise. Our review addressed this gap by detailing common noise sources (e.g., baseline drift, power-line interference, etc.), reviewing denoising techniques (e.g., wavelet transforms, adaptive filtering, autoencoders, etc.), and discussing their downstream impact on model performance. This treatment of ECG signal quality was absent or only marginally addressed in existing reviews.

8. Bias, Fairness, and Equity

We analyzed demographic, algorithmic, and labeling biases, providing a typology of fairness challenges and offering audit strategies for the equitable deployment of AI-ECG systems—an emerging concern in AI ethics scarcely addressed in earlier literature.

9. Clinical Visualization and Recommendations

Unlike predominantly technical reviews, we incorporated visual summaries and tables with actionable insights for both clinical and engineering audiences, bridging the translational gap between research and practice.

10. Integrative Synthesis Across Domains

Our review unified technical, clinical, methodological, and ethical dimensions into a coherent narrative. It highlighted convergence and divergence across studies and provided concrete recommendations for research, development, and clinical translation.

In summary, this review contributes a uniquely integrative, clinically grounded, and methodologically rigorous synthesis of AI-ECG research. It aggregates current knowledge, identifies persistent limitations, and outlines actionable pathways for the safe, effective, and equitable integration of AI technologies in cardiovascular care.

## 5. Conclusions and Recommendations

This comprehensive review synthesizes and critically appraises over 100 recent studies on the application of AI in electrocardiography for detecting CVDs, including HF, MI, AF, stress, and cardiac anomalies. The evidence demonstrated that AI, particularly DL and ML models, consistently outperforms traditional ECG interpretation methods in terms of diagnostic accuracy, risk prediction, and signal processing.

The hallmark of this review was its integrative approach. Its major elements were as follows:

- Broad Coverage of Applications: It offered a detailed classification of AI-ECG applications by disease, model architecture, and methodology, explicitly highlighting the convergences (e.g., the dominance of CNN-based models for arrhythmia and HF detection) and divergences (e.g., the impact of dataset diversity and model explainability) in the literature.
- Comparative Analyses: Through comparative tables and conceptual figures, this review highlighted regularities in performance metrics, methodological trends, and points of agreement among leading research groups, providing a practical resource for clinicians, engineers, and policymakers.
- Multidisciplinary Perspective: In contrast to previous reviews, which often focus narrowly on arrhythmia detection ELMs or synthetic data, this work delivered a broad, up-to-date, and critical synthesis encompassing clinical, technical, and implementation perspectives. This breadth is essential for understanding the real-world challenges and opportunities related to AI-ECG integration.

Our key findings included:

- Superior Diagnostic Performance: DL models such as DenseNet-121, CNN and CNN-BiLSTM achieve accuracy, sensitivity, and specificity rates of >0.96 for coronary artery disease [37]. Similarly, state-of-the-art DL algorithms for arrhythmia detection surpass ML methods in terms of F1-score [25,27].
- Advances in Signal Processing: AI-based denoising preserves ECG morphology, improves signal quality, and outperforms classic filtering techniques, facilitating accurate interpretation even with ambulatory data and enabling more robust downstream analysis [8,47,51,52,55].
- Emerging Applications: The use of AI for stress detection and anomaly identification in real-life settings, as well as the generation of synthetic ECG data via GANs, VAEs, and diffusion models, is expanding the clinical and research scope of ECG analytics [48,110]. Continuous monitoring of stress through wearables and personalized anomaly detection systems represents a frontier that could transform preventive care [75,110].
- Explainability and Fairness: The integration of XAI tools (e.g., saliency maps, Grad-CAM, etc.) is improving transparency and clinical trust, but persistent dataset bias and lack of demographic diversity threaten generalizability and equity [7,61]. Without deliberate efforts to include diverse populations and to provide interpretability, the full benefits of AI-ECG may not be realized for all patient groups [66].
- Implementation Barriers: Real-world deployment is hindered by limited external validation, regulatory uncertainty, and challenges in EHR integration and clinician training. Many AI-ECG algorithms, though promising in research, have yet to be tested in prospective clinical trials or integrated into routine workflows [7,64].

For enhancing the translational impact of our findings and promoting collaborative advancement in the AI-ECG field, we offer the following recommendations:

1. Standardize and Diversify Datasets

Clinicians: They should participate in and advocate for inclusive, multiethnic ECG data collection initiatives, particularly in underrepresented regions, such as Latin America, Africa, and parts of Asia. They can also facilitate demographic metadata recording during ECG acquisition to ensure completeness.

Developers: They should design pipelines that accommodate and test models on diverse datasets, with built-in fairness metrics to assess demographic bias. In addition, they should contribute to the curation of open-access ECG repositories with standardized formats.

Regulators: They should establish minimum dataset diversity requirements for model approval and publication. Furthermore, they should define clear reporting standards for demographic variables and data provenance.

2. Advance Explainability

Clinicians: They should demand transparent models with interpretable outputs that align with clinical reasoning, especially for decision support tools used in critical care. In addition, they should provide feedback on explainability interfaces and usability.

Developers: They should prioritize the integration of XAI techniques (e.g., saliency maps, attention mechanisms, feature attribution, etc.) and validate explanations through clinician feedback. Their research should focus on creating models that balance performance and interpretability.

Regulators: They should require demonstration of model explainability as part of clinical evaluation and consider mandating XAI audits in regulatory submissions for clinical AI tools.

3. Promote Hybrid and Multimodal Models

Clinicians: They should encourage collaborative research that integrates ECG with other diagnostic modalities (e.g., echocardiography, clinical history, biomarkers, etc.). They should also identify clinical scenarios where multimodal approaches could improve diagnosis or triage.

Developers: They should innovate architectures capable of fusing multimodal data (e.g., CNN–recurrent neural network hybrids or transformer-based models) and benchmark their performance against unimodal baselines. They should also publish model ablation studies to demonstrate the added value of each modality.

Regulators: They should develop guidelines on the validation of multimodal models, particularly when different data sources are governed by different privacy and interoperability standards.

4. Strengthen Regulatory and Validation frameworks

Clinicians: They should lead or participate in prospective, multicenter clinical trials evaluating AI-ECG tools, and help define relevant clinical endpoints and usability outcomes.

Developers: They should engage in early dialogue with regulators to understand the requirements for real-world evidence and adaptive model approval, and implement model tracking tools for postmarket surveillance.

Regulators: They should co-create existing guidelines with technical and clinical experts to evaluate AI-ECG tools across the full lifecycle—from development to deployment. Special attention should be given to handling updates in adaptive learning models.

5. Facilitate Continuous Learning and Feedback

Clinicians: They should actively monitor and report the real-world performance of AI-ECG tools, and establish feedback loops to flag false predictions and provide clinical context that can inform model retraining.

Developers: They should implement secure, privacy-preserving continuous learning frameworks (e.g., FL or continual learning with drift detection) to update models safely without compromising generalizability.

Regulators: They should define thresholds for model drift and performance degradation that would trigger mandatory retraining or reapproval. They should encourage the development of audit trails and version control mechanisms in clinical AI software.

Finally, AI has the potential to transform ECG-based cardiovascular care, but realizing it requires interdisciplinary collaboration, methodological rigor, and a commitment to transparency and equity. The findings of this review indicated that technical innovation alone is not enough; issues of data quality, fairness, and user trust must be addressed in parallel. If the community can meet these challenges, AI-augmented ECG interpretation might soon become a ubiquitous part of clinical practice, improving outcomes through earlier detection, more precise risk stratification, and personalized patient management. This review serves as a synthesis of current knowledge and a roadmap for future innovation in AI-driven cardiac diagnostics.

## Figures and Tables

**Table 1:** AI approaches for MI/OMI: Methodological convergences.

Ref.	Model/Method	Sensitivity	Notable Features	Consensus Points
[4]	Rule-based + AI	43.6% (STEMI)	Misses > 50% occlusions	Need for OMI paradigm
[18]	CNN + Imaging	>70%	Multimodal, prehospital ECG	Multimodal fusion improves outcome
[9]	Subtraction ECG	–	Serial comparison, early Dx	Dynamic ECG analysis is superior
[29]	ML, context-free	–	Context-independent detection	AI outperforms classic rules

AI, artificial intelligence; MI, myocardial infarction; OMI, occlusion myocardial infarction; CNN, convolutional neural network; ECG, electrocardiogram; ML, machine learning; STEMI, ST-segment elevation myocardial infarction.

**Table 2:** AI models for HF/LVEF: Performance and dataset characteristics.

Ref.	Model	Dataset/ Size	Internal AUC	External AUC	Key Notes
[1]	DenseNet-121	136,775/ ECG–echo	0.965	0.848	Large single-center dataset
[37]	CNN-BiLSTM	67,332/ ECG	0.943	0.867	Uncertainty estimation integrated
[38]	CNN-BiLSTM	145,220/ ECG	0.958	0.823	Large-scale dataset withuncertainty

AI, artificial intelligence; HF, heart failure; LVEF, left ventricular ejection fraction; CNN, convolutional neural network; LSTM, long short-term memory network; AUC, area under the curve; ECG, electrocardiogram.

**Table 3:** Comparison of noise reduction capabilities of signal processing methods: Regularities and outcomes.

Methodology	Dataset	SNR Improvement	Key Authors	Consensus/Notes
Wavelet (WT) + ACF	MIT-BIH	+6.5 dB	[9]	Best for muscle noise, wearable data
DP-IDAE	MIT-BIH, PTB-XL	+7.06 dB	[8]	Outperforms FIR, WT; preserves shape
Classic FIR/WT	MIT-BIH	<1 dB	(Traditional)	Inferior to AI-based methods

SNR, signal-to-noise ratio; ACF, autocorrelation function; DP-IDEA, dual-path interactive denoising autoencoder; FIR, finite impulse response; AI, artificial intelligence.

**Table 4:** Explainability and fairness: Key findings and recommendations.

Issue	Consensus in Literature	Recommendations from Review
Lack of transparency	XAI improves trust (e.g., saliency maps) [15,44]	Integrate XAI in all clinical, AI-ECG deployments
Dataset bias	Major bias noted [4], underreporting of diversity [13]	Mandate diversity reporting, open data access
Limited accessibility	<30% public datasets available [12]	Standardize metadata, build public ECG repositories

XAI, explainable artificial intelligence; AI, artificial intelligence; ECG, electrocardiogram.

**Table 5:** Comparison of major international reviews on AI and ECG.

Ref	SYS	N	DIS	CMP	INT	SYN	WRB	BIA	VIS	REC	INT
[6]	❌	NS	CVD	❌	❌	❌	❌	❌	❌	❌	❌
[7]	✅	9	AF	✅	❌	❌	✅	❌	✅	✅	❌
[12]	✅	70	⚠️️	⚠️	⚠️	✅	❌	⚠️	❌	⚠️	❌
[15]	✅	50+	CVD	✅	✅	❌	❌	✅	✅	⚠️	⚠️
[16]	✅	80	NS	✅	⚠️	❌	❌	❌	❌	❌	❌
[21]	✅	27	AF	✅	❌	❌	✅	❌	✅	⚠️	❌
[22]	❌	NS	CVD	❌	⚠️	❌	⚠️	❌	❌	⚠️	❌
[23]	❌	NS	CVD	❌	⚠️	❌	❌	❌	❌	⚠️	❌
[26]	❌	NS	⚠️	❌	❌	❌	❌	❌	❌	❌	⚠️
[43]	✅	51	AF	⚠️	❌	❌	⚠️	❌	✅	⚠️	⚠️
[40]	✅	95	HF	✅	⚠️	❌	❌	⚠️	✅	✅	✅
[29]	❌	NS	HF	❌	❌	❌	✅	⚠️	⚠️	✅	⚠️
[24]	❌	NS	CVD	❌	❌	❌	✅	❌	⚠️	⚠️	⚠️
[25]	✅	>100	ARR	✅	⚠️	❌	⚠️	✅	✅	✅	✅
[27]	✅	>100	CVD	⚠️	⚠️	❌	✅	✅	✅	✅	✅
[45]	✅	94	CVD	✅	✅	❌	⚠️	✅	✅	✅	✅
[64]	✅	76	MUL	✅	✅	❌	✅	✅	✅	✅	✅
[66]	✅	>100	CVD	✅	✅	✅	⚠️	✅	✅	✅	✅
[67]	✅	>100	MUL	✅	✅	❌	✅	✅	✅	✅	✅
[71]	✅	NS	MUL, VT	✅	✅	❌	✅	✅	✅	✅	✅
[73]	✅	>100	ST	✅	✅	❌	✅	⚠️	✅	✅	⚠️
[74]	❌	NS	NS	❌	⚠️	❌	✅	✅	❌	✅	⚠️
[77]	❌	–	AD	⚠️	✅	✅	❌	❌	⚠️	⚠️	❌
[81]	✅	60	MUL	✅	⚠️	❌	⚠️	✅	⚠️	✅	⚠️
[87]	✅	65	NS	✅	⚠️	✅	⚠️	⚠️	✅	✅	❌
[88]	✅	82	NS	✅	❌	✅	⚠️	⚠️	✅	✅	❌
[95]	✅	88	MUL	✅	✅	❌	⚠️	✅	✅	✅	⚠️
[91]	⚠️	NS	NS	⚠️	❌	✅	⚠️	✅	⚠️	✅	❌
[92]	✅	NS	NS	⚠️	❌	✅	❌	⚠️	⚠️	✅	❌
[93]	✅	NS	NS	⚠️	❌	✅	❌	✅	⚠️	✅	⚠️
[101]	❌	NS	MUL	⚠️	✅	⚠️	✅	✅	⚠️	✅	⚠️
[76]	❌	NS	ARR	❌	✅	❌	❌	⚠️	❌	❌	❌
[106]	❌	NS	MULT	❌	⚠️	❌	✅	⚠️	⚠️	❌	❌
[107]	✅	67	ARR	✅	⚠️	❌	❌	❌	✅	❌	❌
[108]	✅	39	MULT	✅	⚠️	❌	⚠️	❌	⚠️	✅	⚠️
[109]	✅	27	ARR	✅	❌	❌	❌	❌	❌	❌	❌
Our	✅	>100	MULT	✅	✅	✅	✅	✅	✅	✅	✅

✅ Yes; ❌ No; ⚠️ Partial, tangential, or not applicable; NS, Not specified. SYS, Systematic method (e.g., PRISMA); N, Number of studies included; DIS, Diseases covered; AD, Anomaly detection; CVD, Cardiovascular disease; ARR, Arrhythmias; VT, Ventricular tachycardia; ST, Segment abnormalities; MUL, Multiple cardiac conditions (AF, Atrial fibrillation, MI, Myocardial infarction, HF, Heart failure); CMP, Model comparisons; INT, Interpretability; SYN, Synthetic ECG signals; WRB, Wearables used; BIA, Bias evaluation; VIS, Comparative visualizations; REC, Clinical recommendations; INT, Integrative synthesis (multiapproach).

## Data Availability

No new data were created or analyzed in this study. Data sharing is not applicable to this article.
